# The epigenetic regulatory mechanisms of ferroptosis and its implications for biological processes and diseases

**DOI:** 10.1002/mco2.267

**Published:** 2023-05-22

**Authors:** Molin Yang, Hanshen Luo, Xin Yi, Xiang Wei, Ding‐Sheng Jiang

**Affiliations:** ^1^ Division of Cardiothoracic and Vascular Surgery Tongji Hospital Tongji Medical College Huazhong University of Science and Technology Wuhan Hubei China; ^2^ Department of Cardiology Renmin Hospital of Wuhan University Wuhan Hubei China; ^3^ Key Laboratory of Organ Transplantation, Ministry of Education; NHC Key Laboratory of Organ Transplantation; Key Laboratory of Organ Transplantation, Chinese Academy of Medical Sciences Wuhan Hubei China

**Keywords:** DNA methylation, epigenetics, ferroptosis, histone modifications, noncoding RNA, RNA m^6^A methylation

## Abstract

Ferroptosis is a form of regulated cell death triggered by the iron‐dependent peroxidation of phospholipids. Interactions of iron and lipid metabolism factors jointly promote ferroptosis. Ferroptosis has been demonstrated to be involved in the development of various diseases, such as tumors and degenerative diseases (e.g., aortic dissection), and targeting ferroptosis is expected to be an effective strategy for the treatment of these diseases. Recent studies have shown that the regulation of ferroptosis is affected by multiple mechanisms, including genetics, epigenetics, posttranscriptional modifications, and protein posttranslational modifications. Epigenetic changes have garnered considerable attention due to their importance in regulating biological processes and potential druggability. There have been many studies on the epigenetic regulation of ferroptosis, including histone modifications (e.g., histone acetylation and methylation), DNA methylation, and noncoding RNAs (e.g., miRNAs, circRNAs, and lncRNAs). In this review, we summarize recent advances in research on the epigenetic mechanisms involved in ferroptosis, with a description of RNA N^6^‐methyladenosine (m^6^A) methylation included, and the importance of epigenetic regulation in biological processes and ferroptosis‐related diseases, which provides reference for the clinical application of epigenetic regulators in the treatment of related diseases by targeting ferroptosis.

## Graphical abstract

Ferroptosis is a form of iron‐dependent regulated cell death driven by lipid peroxidation. Current studies have revealed that iron metabolism, lipid metabolism, and redox system jointly regulate ferroptosis. In this review, we highlight epigenetic mechanisms including histone modifications, DNA methylation, noncoding RNAs, and RNA m^6^A modifications in ferroptosis regulation, which provides potential therapeutic targets for ferroptosis‐related diseases.

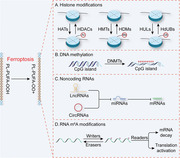

## 1 INTRODUCTION

Ferroptosis is a type of programmed cell death that was discovered in 2012.^1^ Different from apoptosis, necrosis, and autophagy, ferroptosis is driven by extensive iron‐dependent lipid peroxidation. Ferroptosis is closely related to cellular metabolism.^2^, ^3^, ^4^, ^5^, ^6^, ^7^, ^8^, ^9^ Disorders of iron metabolism and lipid metabolism can disrupt the balance between the production and degradation of intracellular reactive oxygen species (ROS), causing ROS accumulation and resulting in cell death. Additionally, studies have pointed out that the occurrence of tumors is often accompanied by the inhibition of ferroptosis, which suggests that multiple proto‐oncogene and tumor suppressor pathways interact to jointly regulate ferroptosis.^10^, ^11^, ^12^, ^13^, ^14^

**FIGURE 1:**
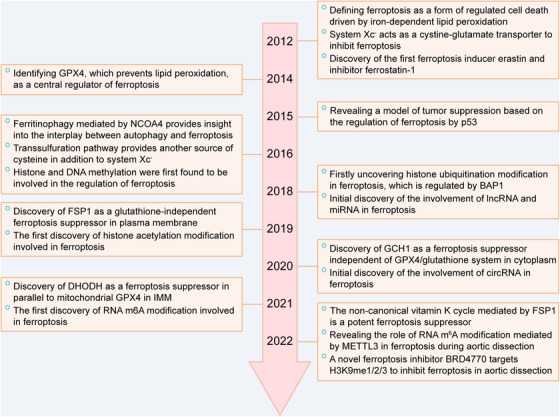
Key discoveries in ferroptosis‐related research. Key molecules related to ferroptosis discovered in different years and the first appearance of different epigenetic modifications targeting ferroptosis. BAP1, BRCA1‐associated protein 1; DHODH, dihydroorotate dehydrogenase; FSP1, ferroptosis suppressor protein 1; GCH1, GTP cyclohydrolase 1; GPX4, glutathione peroxidase 4; METTL3, methyltransferase 3; NCOA4, nuclear receptor coactivator 4; IMM, inner mitochondrial membrane.

Epigenetic regulation refers to a model of regulation that can affect gene expression or activity but does not involve changes to the DNA sequence.^15^ It is involved in the regulation of gene expression through the mediation of transcriptional and posttranscriptional processes. Histone modifications, DNA methylation, and certain noncoding RNAs (ncRNAs) are mainly involved in transcriptional regulation, while microRNAs (miRNAs) are mainly involved in the regulation of posttranscriptional processes.^15^ During epigenetic regulation, four types of regulators “writers,” “erasers,” and “readers,” which are critical for the addition, removal and recognition of epigenetic marks, respectively, and “remodelers,” which moderate the chromatin state, make epigenetic modifications dynamic and reversible.^16^ Increasing evidence suggests that the expression of ferroptosis‐related genes (FRGs) is regulated not only by canonical signaling pathways but also by epigenetic mechanisms.^17^ This review summarizes the progress of ferroptosis study focusing on the epigenetic regulatory mechanisms such as histone modifications, DNA methylation, ncRNAs, and RNA m^6^A modification, highlighting the role of epigenetic regulators in biological processes and ferroptosis‐related diseases therapy (Figure 1).

## 2 THE BASIS OF FERROPTOSIS

As a form of novel programmed cell death, the execution of ferroptosis is driven by iron‐dependent lipid peroxidation. The core events leading to cell death in ferroptosis are considered to be the accumulation of lipid peroxides and iron‐dependent ROS. In this part, the focus is on cellular metabolism, including iron and lipid metabolism, as well as some classical signaling pathways related to ferroptosis.

### 2.1 Iron metabolism during ferroptosis

Ferroptosis is an iron‐dependent form of programmed cell death. Iron molecules with redox activity in cells constitute the “labile iron pool (LIP)” and is called “ferrous iron (Fe^2+^)”,^18^ and their accumulation is a necessary condition of ferroptosis. Mechanistically, the LIP catalyzes the formation of hydroxyl radicals and hydroxides from hydrogen peroxide via the nonenzymatic Fenton reaction. Iron chelators such as dexrazoxane, deferoxamine, and ciclopirox inhibit ferroptosis by binding Fe^2+^.^19^, ^20^ In addition to the Fenton reaction, Fe^2+^ participates in the production of ROS, serving as the cofactor of iron‐dependent enzymes, which are required for the lipid peroxidation in ferroptosis.^3^ Therefore, we deduce that the sources, transport mechanisms, storage modalities, and utilization of iron all affect the ferroptosis sensitivity of cells.

**FIGURE 2:**
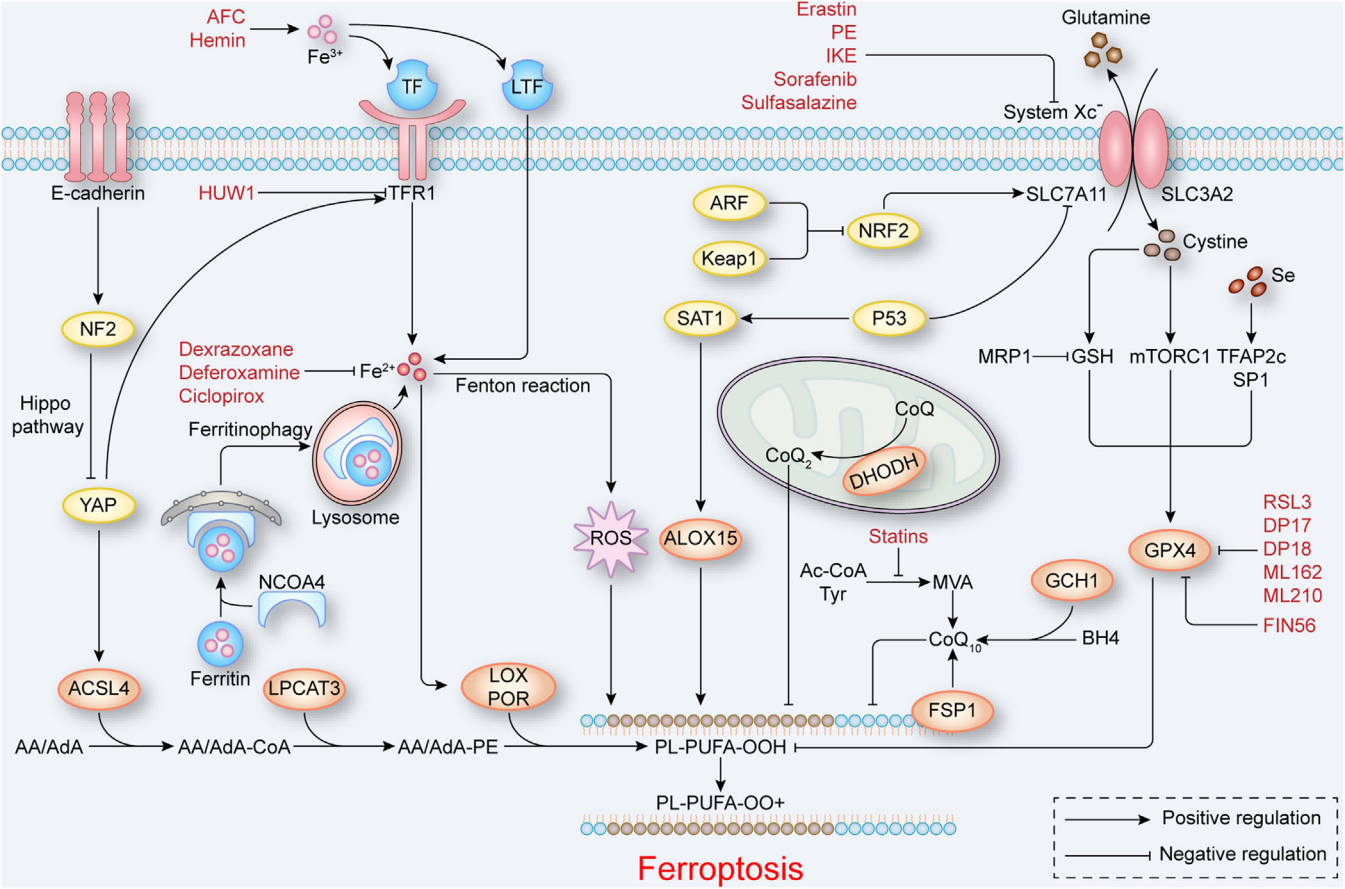
The core mechanisms of ferroptosis. TF, LTF, TFR1, and ferritin are critical for maintaining the balance of intracellular ferrous and regulating the production of ROS and the activity of LOX and POR. System Xc^−^ mediates cysteine intake, GSH synthesis and GPX4 activation, inhibiting lipid peroxidation. Simultaneously, FSP1 and BH4 promote the production of CoQ10, and DHODH promotes the production of CoQ2, and they both protect cells from ferroptosis. P53 promotes and NRF2 inhibits ferroptosis by regulating the expression of SLC7A11, and E‐cadherin inhibits the expression of TFR1 and ACSL4 through the NF2‐YAP signaling pathway to inhibit ferroptosis. ACSL4 catalyzes AA/AdA to generate AA/AdA‐CoA. Subsequently, LPCAT3 catalyzes AA/AdA‐CoA to generate AA/AdA‐PE. Finally, LOX and POR catalyze AA/AdA PE to generate PL‐PUFA‐OOH and induce ferroptosis. The red text indicates ferroptosis regulators. AA/AdA, arachidonic acid/adrenic acid; ACSL4, acyl‐CoA synthetase long‐chain family member 4; BH4, tetrahydrobiopterin; CoA, coenzyme A; CoQ2, coenzyme Q_2_; CoQ10, coenzyme Q_10_; DHODH, dihydroorotate dehydrogenase; FSP1, ferroptosis suppressor protein 1; GPX4, glutathione peroxidase 4; GSH, glutathione; LOX, lipoxygenase; LPCAT3, lysophosphatidylcholine acyltransferase 3; LTF, lactotransferrin; NF2, neurofibromin 2; NRF2, nuclear factor erythroid 2‐related factor 2; PE, phosphatidylethanolamine; PL‐PUFA‐OOH, oxidized PUFA‐containing phospholipids; POR, cytochrome P450 oxidoreductase; ROS, reactive oxygen species; TF, transferrin; TFR1, transferrin receptor 1; YAP, yes‐associated protein 1.

Iron is found as two ionic forms in cells: ferrous (Fe^2+^) and ferric (Fe^3+^). Endogenous iron is derived from hemoglobin iron (Fe^2+^) released by aging red blood cells. Heme oxygenase 1 (HMOX1) catalyzes the release of Fe^2+^ from heme, which provides a major intracellular source of iron, and maintains cellular iron homeostasis.^21^ Overexpression of HMOX1 can induce ferroptosis by the accumulation of Fe^2+^ released from heme,^19^, ^22^ which indicates that there is a beneficial threshold of HMOX1 expression. Iron derived from food takes two forms: nonheme iron (Fe^3+^), which needs to be reduced to Fe^2+^ to be absorbed in the body, and heme iron (Fe^2+^), which can be directly absorbed by intestinal mucosal cells without being complexing cofactors. Ammonium ferric citrate and hemin promote erastin/FINO2‐induced ferroptosis by increasing the sources of iron.^22^, ^23^ Regarding the transport of iron, transferrin (TF) binds to Fe^3+^, which is in nontoxic form, and induces ferroptosis via its iron‐loading capacity.^4^ Moreover, lactotransferrin (LTF), an iron‐binding transport protein, acts like TF to promote ferroptosis by directly increasing iron intake.^24^ However, a recent cell experiment revealed that the lipogenic regulator SREBP2 inhibited ferroptosis by increasing TF levels in circulating melanoma cells.^25^ A similar result of an animal experiment has also been reported. In *Tf*‐knockout mice, exogenous TF protected against ferroptosis induced by a high‐iron diet.^26^ These paradoxical results indicated that the role of TF in ferroptosis might depend on the balance between TF‐bound iron and non‐TF‐bound iron. Ferritin is the main storage form of intracellular iron. Evidence shows that hypoxia increases ferritin‐based storage of Fe^3+^ and reduces the LIP, which leads to greater resistance to ferroptosis.^27^ In contrast, cellular processes that reduce ferritins, such as autophagy, release Fe^2+^, which is the substrate of the Fenton reaction, increasing cell sensitivity to ferroptosis.^5^, ^28^ This selective form of autophagy targeting ferritin is named as ferritinophagy, which is mediated by nuclear receptor coactivator 4 (NCOA4).^29^ In this process, NCOA4 acts as a selective cargo receptor to bind with ferritin and delivers it for lysosomal degradation, finally leading to ferroptosis by the increase of LIP. The novel ferroptosis inhibitor 9a exactly targets NCOA4 to disrupt the interaction between NCOA4 and ferritin, reducing intracellular Fe^2+^.^30^ Additionally, the roles of TF and ferritin are not isolated. The link between TF and ferritin depends on a membrane protein named transferrin receptor 1 (TFR1). Extracellular iron binds with TF, and accumulate within cells in the form of ferritin via TFR1.^31^ Obviously, regulation which targets TFR1 has been associated with ferroptosis. Specifically, TFR1 enhanced ferroptosis induced by ferritinophagy, and in erastin‐treated wild‐type fibroblasts, ferritinophagy led to enhanced TFR1 expression.^28^ In addition, HUW1, a ubiquitin E3 ligase, has been confirmed to be a newly discovered inhibitor of ferroptosis by targeting the degradation of TFR1 (Figure 2).^32^ As previously mentioned, iron is an important cofactor. In ferroptosis, iron is a cofactor of lipoxygenase (LOX) and cytochrome P450 oxidoreductase (POR), which have been identified as iron‐dependent enzymes that drive ferroptosis.^3^ Notably, recent study suggested that POR is more likely than LOX to play a dominant role in ferroptosis.^2^

In conclusion, the imbalance of cellular iron homeostasis, especially the accumulation of intracellular Fe^2+^, can induce ferroptosis. Therefore, reducing the concentration of intracellular Fe^2+^ can effectively inhibit ferroptosis, and maintaining iron homeostasis can be a potential therapeutic strategy to prevent or treat ferroptosis‐related diseases.

### 2.2 Lipid metabolism during ferroptosis

It has been reported that ferroptosis proceeds in two steps: initiation and amplification. Initiation, which involves the selection of substrates, involvement of enzymes and disruption of antioxidant systems, depends on the lipid peroxides produced due to LOX and POR action, whereas amplification, mediated by the LIP, depends on the spread of peroxides across the membrane.^33^ Here we introduce lipid metabolism mainly in the context of ferroptosis initiation.

With respect to substrates, polyunsaturated fatty acids (PUFAs) have been identified as the lipids most prone to peroxidation during ferroptosis due to their bis‐allylic sites.^34^ Moreover, monounsaturated fatty acids (MUFAs) do not readily undergo peroxidation due to the lack of bis‐allylic sites and have been demonstrated to be ferroptosis inhibitors.^34^ Ultimately, acyl‐CoA synthetase long‐chain family member 3 (ACSL3) and stearoyl‐CoA desaturase 1 (SCD1) increase cellular resistance to ferroptosis in a MUFA‐dependent manner.^35^, ^36^ Further study has shown that phosphatidylethanolamine (PE) with arachidonic acid (AA) or adrenic acid (AdA) chains is the most critical substrate involved in ferroptosis.^37^ In addition, oxidized PUFA‐containing phospholipids (PL‐PUFA‐OOH), not free oxidized PUFAs (PUFA‐OOH), cause ferroptosis,^37^ which means that free PUFAs must be esterified to form membrane phospholipids. This process requires the participation of enzymes such as ACSL4 and lysophosphatidylcholine acyltransferase 3 (LPCAT3).^38^ ACSL4 catalyzes the formation of AA/AdA‐CoA to drive free PUFAs into a readily oxidizable state, and LPCAT3 then catalyzes phospholipid remodeling to form AA/AdA‐PE.^39^

The antioxidant systems are key to protect cells from ferroptosis, and its disruption leads to the accumulation of peroxidized phospholipids, which in turn causes ferroptosis. GPX4, a key antioxidant in ferroptosis, can reduce the formation of phospholipid hydroperoxides and thus has been identified as an inhibitor of ferroptosis.^6^ Obviously, targeting GPX4 effectively regulates ferroptosis. Some small‐molecule compounds such as RSL3, DP17, DP18, ML162, and ML210, have been identified as ferroptosis inducers because they directly or indirectly inhibit the activity of GPX4.^40^ GPX4 is essentially a selenoprotein whose molecular structure contains selenocysteine, in which the sulfur in cysteine is replaced with selenium. The utilization of selenocysteine by GPX4 increases its antiferroptic effect.^41^ Thus, the regulation of ferroptosis by selenium is clear. Studies have shown that selenium supplementation can effectively increase ferroptosis resistance in cell and animal models.^41^, ^42^ At the transcription level, selenium may increase the expression of GPX4 in neurons by coactivating the transcription factors TFAP2c and SP1.^42^ In addition to selenium, GSH, a substrate that provides reducing equivalents for GPX4, is integral to the antioxidant effect of GPX4.^43^ Clearly, GSH deficiency increases ferroptosis susceptibility. A genome screen performed for identifying regulators of ferroptosis found that multidrug resistance protein 1 (MRP1), which mediates GSH efflux, increased the sensitivity of tumor cells to ferroptosis by pumping out GSH.^44^ GSH is synthesized from cysteine,^45^ which can be derived from glutamate‐cystine transport system Xc^−^ or transsulfuration cysteine biosynthesis pathway. System Xc^−^, which is composed of a light chain subunit encoded by *SLC7A11* and a heavy chain subunit encoded by *SLC3A2*, exchanges extracellular cystine with intracellular glutamate and cystine is reduced to cysteine to promote the synthesis of GSH.^46^ Indeed, the first ferroptosis inducer to be identified, erastin, was found to mainly inhibit system Xc^−^, resulting in the depletion of GSH and a reduction in GPX4 activity.^1^ Additional system Xc^−^ inhibitors have subsequently discovered, such as piperazine erastin, imidazole ketone erastin, sorafenib, and sulfasalazine.^40^ In addition to promoting GSH synthesis, system Xc^−^‐mediated cystine transport promotes GPX4 synthesis. Mechanistically, mechanistic target of rapamycin complex 1 (mTORC1) couples cystine uptake with GPX4 synthesis, and cystine may facilitate GPX4 synthesis partly through the Rag‐mTORC1‐4EBP signaling axis in a GSH‐independent way.^47^ However, the mechanism through which cystine recognizes mTORC1 remains to be further investigated. As another source of cysteine, transsulfuration cysteine biosynthesis pathway is an alternative antioxidant process when system Xc^−^ is inhibited. There are two key enzymes cystathionine β‐synthase (CBS) and cystathionine γ‐lyase (CTH) in the pathway. CBS catalyzes the synthesis of cystathionine with methionine cycle intermediate homocysteine as the substrate, and then cystathionine can be cleaved by CTH to release cysteine.^48^ In prolonged erastin‐treated cells, transsulfuration cysteine biosynthesis pathway was found to be continuously activated since the upregulation of CBS, which confers erastin‐induced ferroptosis resistance.^49^ Additionally, the knockdown of cysteinyl tRNA synthetase 1 (CARS), an enzyme that links cysteine with tRNAs for protein translation, inhibits erastin‐induced ferroptosis by the upregulation of transsulfuration genes including *CBS*, *CTH*, *PSAT1*, and *PSPH*.^50^

In ferroptosis in addition to GPX4, nonmitochondrial coenzyme Q_10_ (CoQ_10_), a major antioxidant system inhibits lipid peroxidation by trapping radical intermediates. CoQ_10_ synthesized in vivo is derived from mevalonate (MVA) generated by tyrosine and acetyl‐CoA through a series of enzymatic reactions. Notably, the synthesis of MVA directly affects the antioxidant capacity of CoQ_10_. Statins, which reduce MVA production by inhibiting HMG‐CoA reductase, sensitizes cells to FIN56, a ferroptosis inducer that promotes GPX4 degradation.^7^ In addition, a recent study has shown that inhibition of the MVA pathway by statins inactivated GPX4 to induce ferroptosis.^51^ This evidence shows that the induction of ferroptosis by statins includes the involvement of both CoQ_10_ and GPX4; therefore, further exploration into whether their effects are mutual or independent is necessary. Studies have shown that ferroptosis suppressor protein 1 (FSP1), which is also called apoptosis‐inducing factor mitochondria‐associated 2, prevent GPX4‐deficient cells from ferroptosis by acting as an NADPH‐dependent CoQ oxidoreductase to regenerate reduced CoQ_10_,^8^, ^9^ which suggests that regulation of ferroptosis by CoQ_10_ is independent of GPX4. Therefore, the NADPH–FSP1–CoQ_10_ ferroptosis pathway was identified as a parallel system to the GSH–GPX4 pathway that regulates ferroptosis. Moreover, a recent study found that noncanonical vitamin K acts as a potential ferroptosis inhibitor through its reducing form hydroquinone (VKH2).^52^ Since the similar structure properties between vitamin K and CoQ_10_, FSP1 was also found to act as a vitamin K reductase. However, in FSP1‐KO cells, high dose of phylloquinone (vitamin K1) and menaquinone‐4 (vitamin K3) can still prevent ferroptosis, which indicates the mechanism of vitamin K cycle preventing ferroptosis still needs further study.^52^ In addition, a recent antagonistic ferroptosis gene screening revealed another independent ferroptosis inhibition pathway, the GCH1–BH4 pathway. GTP cyclohydrolase 1 (GCH1) is the rate‐limiting enzyme of tetrahydrobiopterin/dihydrobiopterin (BH4/BH2) production, and BH4 promotes the production of reduced CoQ_10_ and selectively inhibits lipid peroxidation.^53^ Notably, as the first enzyme that catalyzes CoQ_10_ biosynthesis, ferroptosis induced by CoQ_2_ deletion was not reversed by FSP1,^9^ but dihydroorotate dehydrogenase (DHODH) in the mitochondrial inner membrane inhibited ferroptosis through this pathway. Mechanistically, DHODH couples the oxidation of DHO with the reduction of CoQ and generates CoQ_2_ in the mitochondrial inner membrane^54^ (Figure 2). Notably, it was found that mitochondrial plays a critical role in cysteine deprivation‐induced ferroptosis, but not in GPX4 inhibition‐induced ferroptosis,^55^ which suggests that the cellular protective system against ferroptosis mediated by DHODH in the mitochondrial inner membrane is independent of GPX4.

In general, the antioxidant system involved in lipid metabolism can be categorized into at least six pathways, namely, the system Xc^−^–GPX4, MVA–CoQ_10_, FSP1‐reduced CoQ_10_, FSP1–VKH2, GCH1–BH4, and DHODH–CoQ_2_ pathways. Many epigenetic mechanisms regulating these pathways have been elucidated,^56^ which are discussed in depth in subsequent subsections.

### 2.3 The signaling pathways in ferroptosis

Inhibiting tumor growth has been shown to be a physiological function of ferroptosis. Several oncogenic and tumor suppressor pathways are involved in ferroptosis. In these pathways, SLC7A11 is considered a central link. The expression of SLC7A11 is regulated by different upstream molecules, such as p53 and NRF2.

The tumor suppressor protein p53 represses the transcription of *SLC7A11*,^10^ and this effect is affected by p53 protein acetylation modification. A study demonstrated that the acetylation‐defective mutant p53^3kR^ (K117R+K161R+K162R) retains the ability to regulate ferroptosis by downregulating SLC7A11 unless the fourth acetylation site K98R is mutated along with other lysine sites.^57^ In addition to affecting GPX4 activity or synthesis through the p53–SLC7A11 axis, downregulation of SLC7A11 by p53 indirectly activates arachidonate 12‐lipooxygenase (ALOX12), which is an ACSL4‐independent pathway.^14^ In addition to *SLC7A11*, p53 regulates other downstream targets. For example, p53 activates the spermidine/spermine N1‐acetyltransferase 1 (*SAT1*) gene, which induces lipid peroxidation to induce ferroptosis. In this process, the expression level of arachidonate 15‐lipoxygenase (ALOX15) is correlated with SAT1 induction.^58^ Additionally, p53 promotes the expression of glutaminase 2 (*GLS2*) gene, which encodes a mitochondrial GLS to produce glutamate from glutamine.^59^ With increasing production of glutamate, GLS2 leads to enhanced mitochondrial respiration and ATP generation by regulating energy metabolism. Ultimately, mitochondrial GLS2, but not cytosolic GLS1, has been confirmed to induce glutaminolysis‐associated ferroptosis.^4^, ^60^ The above evidence supports the promoting effect of p53 on ferroptosis. However, p53 can also inhibit ferroptosis in a transcription‐independent way. In colorectal cancer, depletion of p53 prevents nuclear accumulation of dipeptidyl‐peptidase‐4 (DPP4), which promotes DPP4‐dependent lipid peroxidation to result in ferroptosis.^61^ The inhibiting effect of p53 on ferroptosis may also be attributed to the calcium‐independent phospholipase iPLA2β. As a direct target of p53, iPLA2β‐mediated lipid detoxification is critical for inhibiting ROS‐induced ferroptosis. Interestingly, iPLA2β is differentially regulated by p53. Under low levels of stress, p53 can active the expression of iPLA2β, but this activation is diminished under high levels of stress.^62^ Overall, p53 plays a dual role in ferroptosis, which needs further study to find out whether cell environment or cell type determines the regulatory effect of p53 on ferroptosis.

In contrast to p53, nuclear factor erythroid 2‐related factor 2 (NRF2) promotes the expression of *SLC7A11*.^63^ Two upstream molecules of NRF2, kelch‐like ECH‐associated protein 1 (KEAP1) and alternative reading‐frame protein (ARF), have also been found to be associated with ferroptosis. The tumor suppressor KEAP1 binds to NRF2 in the cytoplasm and degrades NRF2 via ubiquitination. Under oxidative stress, NRF2 dissociates from KEAP1, is translocated to the nucleus, where it binds to the antioxidant response element to activate the transcription of downstream genes,^11^, ^64^ such as *SLC7A11*. ARF regulates NRF2 in a KEAP1‐independent manner and inhibits CBP‐dependent NRF2 acetylation to inhibit NRF2 transcriptional activity, ultimately promoting ferroptosis.^12^

Both p53 and NRF2 mediate intracellular signal transduction. Moreover, a ferroptosis‐regulating pathway that depends on intercellular signaling has been identified in epithelial cells. Intercellular communication is mediated by E‐cadherin, which activates the intracellular Hippo pathway to negatively regulate the proto‐oncogenic transcriptional coactivator YAP. The NF2‐YAP pathway ultimately downregulates the expression of ACSL4 and TFR to inhibit ferroptosis (Figure 2).^13^ Mesenchymal or metastatic cancer cells have been shown to be sensitive to ferroptosis‐inducing agents,^65^ possibly because of decreased cadherin activity during the epithelial–mesenchymal transition (EMT).

In conclusion, iron metabolism, lipid metabolism, and tumor‐related signaling pathways interact with each other during ferroptosis, and lipid metabolism can be regarded as the core link among these pathways. Perturbed iron metabolism disrupts the dynamic balance of ROS generation and elimination, which in turn causes lipid oxidative stress. Fe^2+^ participates in this stress‐including process as cofactors for enzymes such as LOX and POR. In addition to regulating antioxidant systems such as GPX4 and system Xc^−^, tumor‐related signaling pathways affect the activity of enzymes required for lipid peroxidation such as ALOX12. The formation of lipid peroxides on the membrane is a result of lipid metabolism in ferroptosis; however, the specific mechanism of ferroptosis induced by membrane lipid peroxides needs to be further studied.

## 3 EPIGENETIC REGULATION IN FERROPTOSIS

Epigenetics refers to heritable changes in gene function that ultimately lead to phenotypic changes without changes in the DNA sequence. Increasing evidence suggest that epigenetic regulation affects ferroptosis through gene transcription, posttranscription, or posttranslation processes, and targeting epigenetic mechanisms in ferroptosis is expected to provide a new direction for the treatment of ferroptosis‐related diseases. In this context, we will focus on the mechanisms of epigenetic regulation including histone modifications, DNA methylation, ncRNAs, and RNA modifications in ferroptosis.

### 3.1 Histone modifications in ferroptosis

Histone modifications regulate gene expression by altering the structural state of chromatin, and they include acetylation, methylation, phosphorylation, adenylation, and ubiquitination. Here, we mainly describe the regulatory mechanisms of acetylation, methylation and ubiquitination in ferroptosis.

#### 3.1.1 Histone acetylation in ferroptosis

**FIGURE 3:**
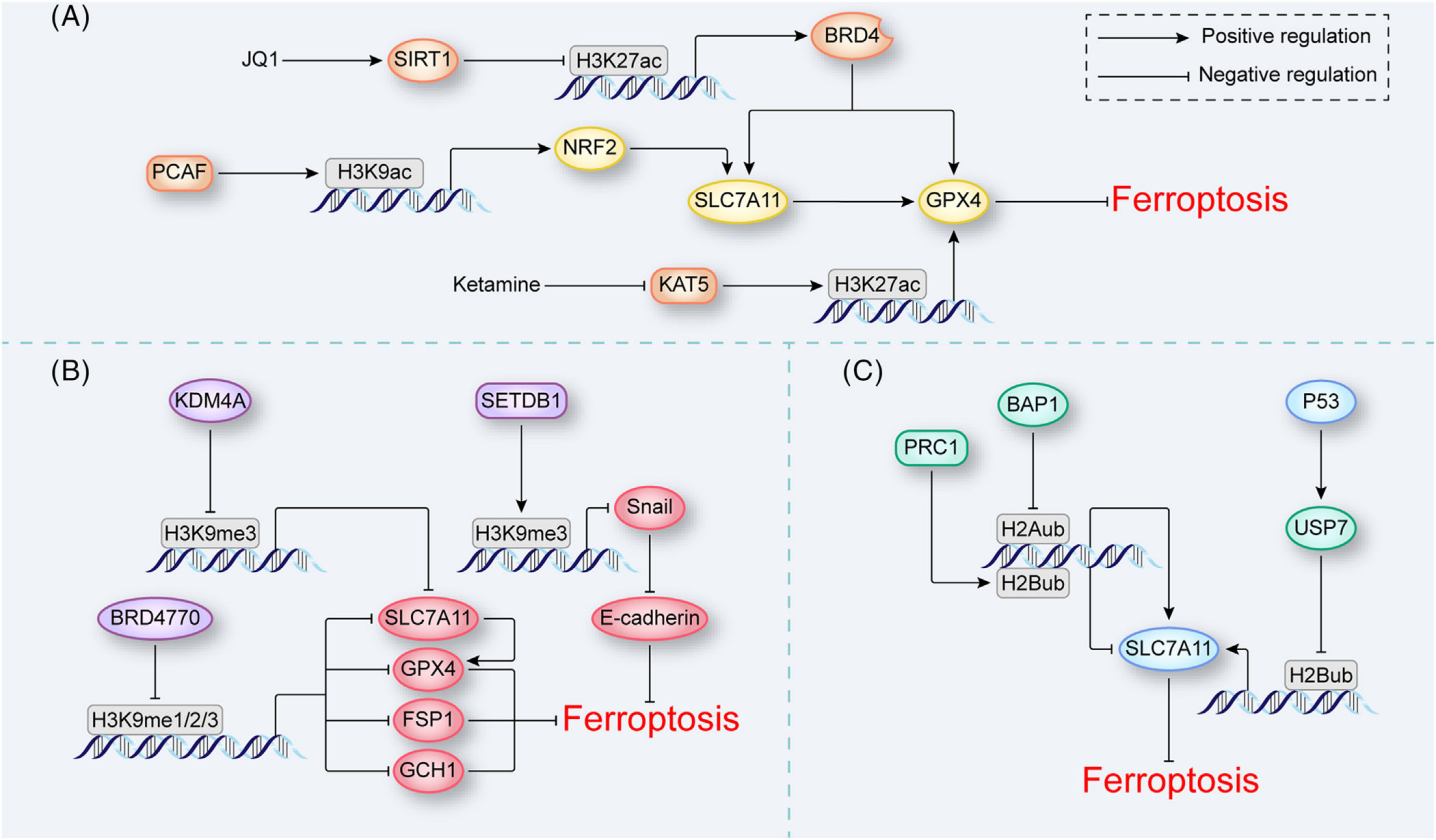
Histone modifications in ferroptosis. (A) Histone acetylation in ferroptosis. JQ1 and ketamine reduce H3K27ac abundance at BRD4 and GPX4 genes to induce ferroptosis, respectively. PCAF increases H3K27ac abundance at NRF2 gene to inhibit ferroptosis. (B) Histone methylation in ferroptosis. KDM4A reduces and SETDB1 increases H3K9me3 abundance at SLC7A11 and Snail genes, respectively, to regulate ferroptosis. BRD4770 reduces H3K9me1/2/3 abundance at SLC7A11, GPX4, FSP1, and GCH1 genes simultaneously to inhibit ferroptosis. (C) Histone ubiquitination in ferroptosis. PRC1 increases and BAP1 reduces the H2Aub abundance at SLC7A11 gene, both proteins induce ferroptosis. P53 increases the expression of USP7, which reduces H2Bub abundance at SLC7A11 gene to induce ferroptosis. BAP1, BRCA1‐associated protein 1; BRD4, bromodomain‐containing protein 4; FSP1, ferroptosis suppressor protein 1; GCH1, GTP cyclohydrolase 1; GPX4, glutathione peroxidase 4; KDM4A, lysine demethylase 4A; NRF2, nuclear factor erythroid 2‐related factor 2; PCAF, P300/CBP‐associated factor; PRC1, protein regulator of cytokinesis 1; SETDB1, SET domain bifurcated 1; USP7, ubiquitin‐specific peptidase 7.

Histone acetylation neutralizes its positive charge and impairs the ability of histones to bind DNA, which leads to depolymerized nucleosomes and the activation of gene transcriptional.^66^, ^67^ Histone acetylation depends on the bromodomain‐containing protein (BRD) family, histone acetyltransferases (HATs), and histone deacetylases (HDACs). The BRD family recognizes acetylation marks.^68^ In cancer cells, the BRD4 inhibitor JQ1 induces ferroptosis by enhancing the expression of an HDAC named SIRT1, which decreases the H3K27ac level at upstream of *BRD4*, ultimately affecting the recognition of acetylation sites on the histones at *GPX4* and *SLC7A11* genes.^69^ Two competing enzymes, HATs and HDACs are critical for regulating histone acetylation.^70^ Histone hyperacetylation by HATs is associated with transcriptional activation. For instance, NRF2 recruits P300 and CBP, and P300/CBP‐associated factor (PCAF) may increase H3K9ac level at NRF2 to regulate ferroptosis in renal tubulointerstitial fibrosis.^71^ In contrast, ketamine, an inhibitor of lysine acetyltransferase 5 (KAT5), reduces H3K27ac levels at *GPX4* promoter regions to promote ferroptosis in breast cancer (Figure 3A).^72^ In addition, in liver cancer, two transcription factors, hepatocyte nuclear factor 4 alpha (HNF4A) and HIC ZBTB transcriptional repressor 1 (HIC1), competitively bind with KAT2B, playing opposite roles regulating GSH production; the former has been identified a ferroptosis inhibitor, and the latter has been identified a ferroptosis inducer.^73^ Similar to HAT inhibitors, histone deacetylation mediated by HDACs also exhibits transcriptional repression. NAD^+^‐dependent HDACs such as SIRT1 and SIRT3 trigger ferroptosis induction by inhibiting EMT markers in cancer cells.^65^, ^74^ Of course, the effect of HDAC is reversed by HDAC inhibitors in the tumor microenvironment. HDAC inhibitors such as BEBT‐908 hyperacetylate *p53* to promote ferroptosis signaling.^75^ Nevertheless, the regulation of ferroptosis by BEBT‐908 may be affected by other signaling pathways such as the PI3K pathway. Other HDAC inhibitors, such as quisinostat and vorinostat, also induce ferroptosis by inhibiting GPX4 and system Xc^−^ expression respectively; however, their targets are not clear.^76^, ^77^ Interestingly, the regulation of ferroptosis in different cells may be opposite even after treatment with the same HDAC inhibitors. A study revealed that in neurons and cancer cells with similar erastin‐induced ferroptosis mechanism, class I HDAC inhibitors enhanced ferroptosis in cancer cells, while protecting neurons from ferroptosis. The cell‐specific biological effects of HDAC inhibitors may result from the differential expression of distinct HDAC8 between cancer cells and primary neurons.^78^ Therefore, it is necessary to further study the cell‐specific expression mechanism of HDAC, which will help us to understand how histone acetylation modifications precisely regulate ferroptosis.

#### 3.1.2 Histone methylation in ferroptosis

Another important form of histone modification, histone methylation, mainly involves modification of an N‐terminal lysine (K) or arginine (R) residue in the H3 or H4 histone.^79^, ^80^, ^81^, ^82^ Generally, different methylation sites and the number of methyl groups on H3 and H4 indicate different biological functions. Among these modifications, H3K4me1/2/3, H3K36me1/2/3, and H3K79me1/2/3 usually mediate transcriptional activation, while H3K9me3, H3K27me3, and H4K20me2/3 usually repress transcription.^82^, ^83^

The histone methylation modifications related to ferroptosis mainly involve methylation of H3K4 and H3K9 catalyzed by histone methyltransferases (HMTs).^84^ Regarding the methylation of H3K4, a study found that GPX4 was more highly expressed in tumor cells than in normal cells partially because of the increased abundance of H3K4me3 at the promoter of *GPX4*.^85^ Additionally, in gastric cancer, methionine adenosyltransferase 2A (MAT2A) promoted the production of the methylation donor SAM, which upregulated ACSL3 by increasing H3K4me3 abundance at the promoter, sequentially inhibiting ferroptosis.^86^ In addition, methylation of H3K4 indirectly regulates ferroptosis by affecting the recognition of histone acetylation sites. JQ1 inhibited the expression of an HMT named G9a, reduced H3K4me3 abundance at *BRD4*, and further induced ferroptosis in cancer cells.^69^ These results suggested that the H3K4me3 modification inhibits ferroptosis in cancer cells. Reasonably, one can assume that the methylation of H3K9 induces ferroptosis, and this outcome has also been confirmed to some extent. In breast cancer, mucin1‐C (MUC1‐C), a transmembrane oncoprotein, bound with the CD44 variant to stabilize the SLC7A11 molecule. In turn, H3K9me2/3 at the *MUC1‐C* promoter inhibited *MUC1‐C* gene transcription, which influenced the ability of GPX4 to induce ferroptosis.^87^ Similar results have been shown not only in tumors but also in progressive diseases. In a pulmonary fibrosis model, SET domain bifurcated 1 (SETDB1) indirectly induced E‐cadherin expression by increasing H3K9me3 abundance at the *Snail* promoter, which in turn enhanced TGF‐β‐induced ferroptosis.^88^ Experiments related to histone demethylases, which are specifically critical for removing methyl groups from modified histones, have further proven this inference. For example, lysine demethylase 4A (KDM4A) protected against ferroptosis by decreasing H3K9me3 abundance at the *SLC7A11* promoter to upregulate the expression of SLC7A11 in osteosarcoma.^89^

These studies on histone methylation were conducted mostly in the context of cancer and degenerative diseases, which have been shown to be closely affected by ferroptosis. Interestingly, the findings of our group and others have suggested that multiple forms of regulated cell death are involved in the development of aortic dissection (AD), and histone methylation plays a critical role in these biological processes.^83^, ^90^, ^91^, ^92^, ^93^, ^94^, ^95^ More importantly, our recently published results revealed that ferroptosis, in particular, is involved in the development of AD.^96^ Furthermore, by screening multiple inhibitors of methyltransferases, we found that BRD4770 negatively regulated ferroptosis in VSMCs by inhibiting the H3K9me1/2/3 modifications. BRD4770 inhibited ferroptosis not only by inducing the expression of classical ferroptosis regulatory genes, such as *FSP1*, *SLC7A11*, *GPX4*, and *GCH1* (Figure 3B), but also by inhibiting inflammation‐related genes activation. Moreover, BRD4770 protected against AD development by inhibiting ferroptosis.^96^ In agreement with those of other studies, our results confirmed that H3K9me3 promotes ferroptosis. However, in clear cell renal cell carcinoma (ccRCC), suppressor of variegation 3−9 homolog 1 (SUV39H1), an HMT that deposits H3K9me3, has been found to protect cells against ferroptosis. Mechanistically, SUV39H1 targets the promoter of dipeptidlypeptidase‐4 (*DDP4*) and inhibits the expression of DPP4, which binds to NADPH oxidase 1 (NOX1) to induce lipid peroxidation.^97^ It seems that the regulation effects of H3K9me3 on ferroptosis differ in different cells; therefore, it is necessary to explore the factors, such as cell microenvironment, which influence the biological effects of histone methylation modification in different cell lines.

#### 3.1.3 Histone ubiquitination in ferroptosis

In cancer cells, current knowledge suggests that the regulation of ferroptosis by histone ubiquitination mainly involves histone 2A ubiquitination (H2Aub) and histone 2B ubiquitination (H2Bub), both of which have been associated with the expression of SLC7A11.^98^, ^99^ The level of the tumor suppressor BRCA1‐associated protein 1 (BAP1) decreases and the ubiquitin ligase of H2Aub named PRC1 increases H2Aub occupancy at the *SLC7A11* promoter. In theory, in contrast to the regulation of H2Aub by BRP1, regulatory effects induced by PRC1 produces different phenotypes; however, both regulatory proteins repressed SLC7A11 expression.^98^, ^100^ This unexpected result suggested that H2Aub itself is not the sole mediator of these regulatory effects; in fact, the homeostasis of H2A ubiquitination and deubiquitination influences H2Aub‐mediated target gene expression. In addition, p53 promotes the recruitment of ubiquitin‐specific peptidase 7 (USP7) and leads to a decrease in H2Bub abundance at the *SLC7A11* gene regulatory region, decreasing the expression of SLC7A11 in a p53 transcription factor‐independent manner (Figure 3C).^99^

In general, histone modifications, such as acetylation, methylation, or ubiquitination, can modulate the susceptibility of cells to ferroptosis by affecting the expression of genes involved in ferroptosis‐related metabolism pathways. However, the effects of histone modifications in ferroptosis can be context dependent, and different patterns of modifications may have opposite or synergistic effects on ferroptotic cells. Further research is needed to fully understand the mechanisms underlying the regulation of ferroptosis by histone modifications.

### 3.2 DNA methylation in ferroptosis

DNA methylation is a common epigenetic modification in eukaryotic cells. In mammals, DNA methylation usually involves SAM as the methyl group donor and is mainly catalyzed by DNA methyltransferases (DNMTs), such as DNMT1, DNMT3A, and DNMT3B, and the DNA methylation level is always negatively correlated with its expression level.^101^

**FIGURE 4:**
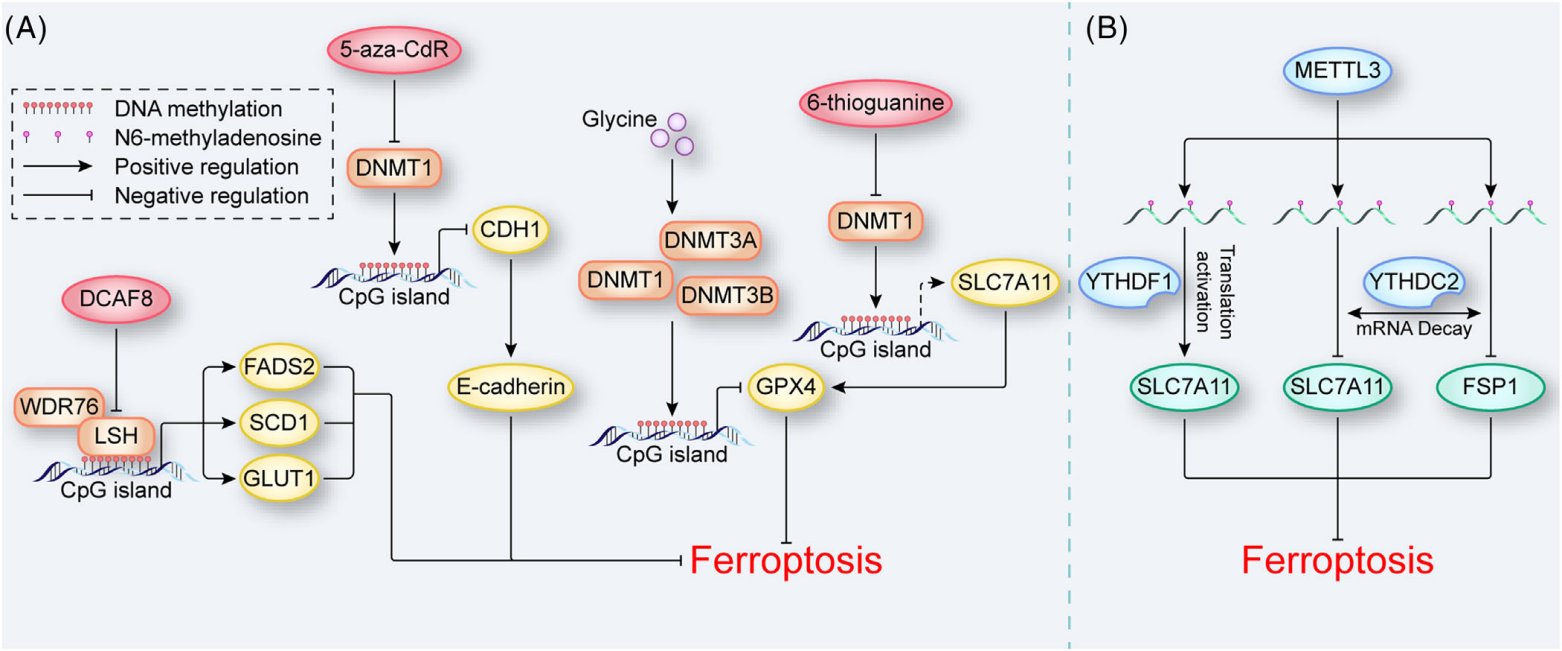
DNA and RNA methylation in ferroptosis. (A) DNA methylation in ferroptosis. LSH interacts with WDR76 to inhibit ferroptosis by regulating DNA methylation, and this effect is antagonized by DCAF8. DNMTs including DNMT1, DNMT3A, and DNMT3B increase the DNA methylation levels of target genes to regulate ferroptosis. In contrast, DNMTs inhibitors, such as 5‐aza‐CdR and 6‐thioguanine, antagonize the biological effects of DNMTs. (B) RNA m6A modification in ferroptosis. METTL3 promotes the m6A modification of SLC7A11 and FSP1 mRNA. YTHDF1 recognizes the m6A marks on SLC7A11 mRNA and increases its translation to inhibit ferroptosis. YTHDC2 recognizes the m6A marks on SLC7A11 and FSP1 mRNA, leading to mRNA decay to induce ferroptosis. The dotted line represents indirect regulation. DCAF8, DDB1‐ and CUL4‐associated factor 8; DNMT, DNA methyltransferase; FSP1, ferroptosis suppressor protein 1; LSH, lymphoid‐specific helicase; METTL3, methyltransferase 3; WDR76, WD repeat domain 76; YTHDC2, YTH domain containing 2; YTHDF1, YTH N6‐methyladenosine RNA binding protein 1.

Evidence suggests that DNA methylation regulates ferroptosis by participating in lipid metabolism. During the generation of substrates, a study revealed that overexpression of elongation of very long‐chain fatty acid protein 5 (ELOVL5) and fatty acid desaturases 1 (FADS1) in mesenchymal‐type gastric cancer cells (GCs), which promote the biosynthesis of PUFAs, was downregulated in intestinal‐type GCs due to the methylation of the *ELOVL5* and *FADS1* promotors.^102^ In addition, in lung cancer, lymphoid‐specific helicase (LSH), a protein belonging to the sucrose nonfermenting 2 family of chromatin‐remodeling enzymes, directly modifies DNA methylation with WD repeat domain 76 (WDR76) to activate metabolic genes, including *GLUT1*, *FADS2*, and *SCD1*, inhibiting ferroptosis by decreasing lipid ROS levels.^103^ The effect of LSH in this process was antagonized by DDB1‐ and CUL4‐associated factor 8 (DCAF8).^104^ DNA methylation also directly regulates the expression of Ferroptosis‐related molecules such as GPX4 and SLC7A11. In rheumatoid arthritis, glycine enhanced SAM‐mediated *Gpx4* promoter methylation catalyzed by DNMT1, DNMT3A, and DNMT3B to induce ferroptosis.^105^ The DNMT1 inhibitor 6‐thioguanine has also been confirmed to be a ferroptosis inducer in gastric cancer, which may be related to its indirect inactivation of system Xc^−^.^106^ In addition, DNA methylation is involved in the regulation of intercellular interactions in ferroptosis. The DNMT1 inhibitor 5‐aza‐CdR decreased the methylation level of cadherin‐1 (*CDH1*) in head and neck cancer, increasing E‐cadherin expression and decreasing ferroptosis sensitivity (Figure 4A).^65^ These findings clearly demonstrated that DNA methylation actively regulates ferroptosis. Notably, oxidative stress and iron metabolism directly affect DNA methylation levels.^107^, ^108^, ^109^, ^110^ A recent study found that chronic iron exposure increased LIP levels in colon cells to promote ferroptosis while reactively triggering demethylation of NRF2 targets such as *NOQ1* and *GPX2*, which are protective factors against ferroptosis and this epigenetic change was time‐dependent and reversible.^111^ This finding suggests that DNA methylation and ferroptosis may be mutually causal, and the determination of whether this causal relationship is involved in other FRGs or epigenetic regulatory effects requires further study.

In summary, DNA methylation plays a critical role in regulating ferroptosis through various mechanisms, including the regulation of lipid metabolism and the modulation of intercellular interactions. Deeply understanding the mechanisms of DNA methylation in ferroptosis is crucial for the development of effective therapies for ferroptosis‐related diseases.

### 3.3 Noncoding RNAs in ferroptosis

In recent years, increasing number of ncRNAs with biological functions have been discovered. NcRNAs can be classified into two main types: constitutive ncRNAs and regulatory ncRNAs. Constitutive ncRNAs act like housekeeping genes in translation and splicing, and they include ribosomal RNAs, transfer RNAs, and small nuclear RNAs. From an epigenetic perspective, regulatory ncRNAs are more interesting because they are involved in the modification of transcriptional and posttranscriptional processes. Here, we mainly describe the roles of certain regulatory ncRNAs in ferroptosis.

#### 3.3.1 miRNAs in ferroptosis

MiRNAs constitute a class of noncoding single‐stranded RNA molecules approximately 22 nucleotides in length and encoded by endogenous genes. MiRNAs regulate gene expression at the mRNA level. Mechanistically, miRNAs participate in epigenetic regulation mainly by binding to the 3′UTR of target sequences, inhibiting mRNA translation or promoting mRNA degradation. The identification of FRGs in pulmonary arterial hypertension led to the construction of a network involving miRNA and transcription factors,^112^ which showed that miRNAs are involved in the regulation of ferroptosis. This regulatory mechanism was confirmed by iron metabolism gene interference therapy based on miRNA for cancers.^113^ Fortunately, a case has been described in which nanomedicine with a miRNA targeting ferroptosis was used to treat tumors in vivo.^114^

The regulation of ferroptosis by miRNAs is not homogeneous but multifaceted and has been document not only in ferroptosis‐related disease models such as tumor, ischemic injury, and degenerative disease models^115^, ^116^, ^117^, ^118^, ^119^, ^120^ but also in normal tissues.^121^, ^122^ Multiple metabolic pathways have been identified as major links between miRNAs and ferroptosis. MiR‐302a‐3p and miR‐335 respectively target ferroportin and ferritin to promote ferroptosis by regulating iron metabolism.^115^, ^117^ Additionally, miR‐30e‐5p targets specificity protein 1 (SP1), which is an important molecule in energy metabolism, to inhibit the AMPK pathway and thus induce ferroptosis.^121^ Regarding classical signaling pathways, miR‐5096, miR‐375, and miR‐378a‐3p downregulate the expression of SLC7A11 to induce ferroptosis,^116^, ^118^, ^123^ in addition, miR‐15a‐5p, miR‐324‐3p, miR‐182‐5p, and miR‐541‐3p promote ferroptosis by inhibiting GPX4 expression.^118^, ^119^, ^124^, ^125^ Additionally, miR‐214‐3p promotes ferroptosis by targeting ATF4,^126^ which is a critical mediator of endoplasmic reticulum (ER) stress.^127^ Notably, ER stress plays a dual role in ferroptosis. In human glioma cells ATF4 acts as a negative ferroptosis regulator by upregulating SLC7A11,^128^ which is consistent with the above result. However, in breast cancer, ATF4 upregulates the expression of ChaC glutathione specific gamma‐glutamylcyclotransferase 1 (CHAC1) to promote the cystine starvation‐induced ferroptosis.^129^

In addition to inducing ferroptosis, certain miRNAs inhibit ferroptosis. During lipid metabolism, miR‐670‐3p and miR‐424‐5p suppress ferroptosis by inhibiting ACSL4 expression.^130^, ^131^ MiR‐16‐92 also causes ferroptosis resistance by inhibiting the expression of zinc lipoprotein A20, which is a molecule upstream of ACSL4.^122^ Similarly, as the upstream molecules of SLC7A11, p53, and NRF2 are regulated by miRNAs. MiR‐122‐5p and miR‐130b‐3p inhibit ferroptosis by targeting p53 and DKK1, respectively, with the latter blocking the NRF2 signaling pathway.^132^, ^133^ In addition, miR‐545, miR‐137, miR‐190a‐5p, miR‐23a‐3p, miR‐212‐5p, and miR‐194 respectively target other metabolism‐related genes *TF*, *SLC1A5, Gls2*, *Dmt1*, *Ptgs2*, and *Bach1*, to inhibit ferroptosis.^120^, ^134^, ^135^, ^136^, ^137^, ^138^

**FIGURE 5:**
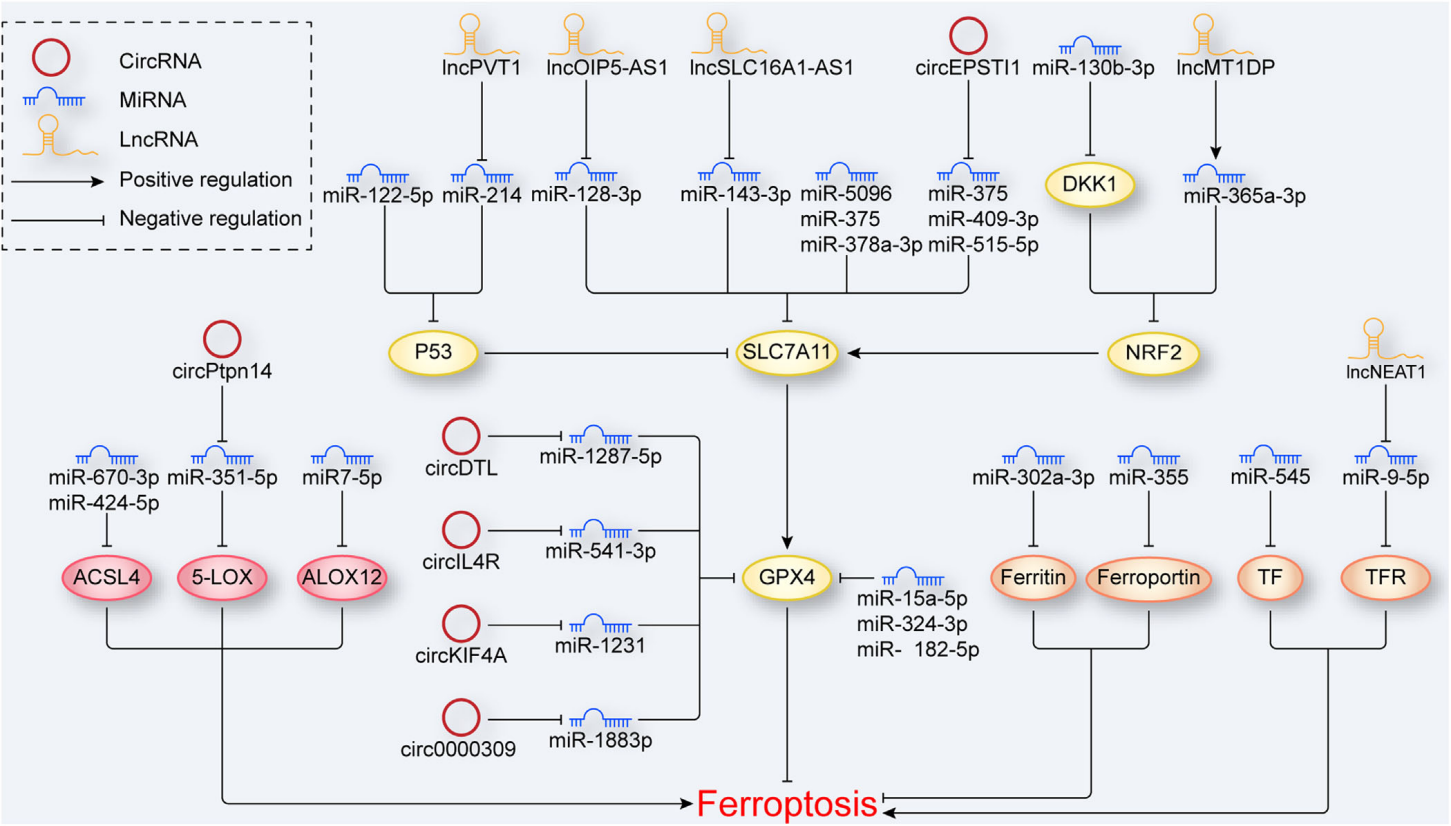
NcRNAs in ferroptosis. In ferroptosis, regulation by ncRNAs can be classified into three types. (1) NcRNAs in lipid metabolism. NcRNAs regulate ferroptosis by targeting lipid metabolism‐related molecules, such as ACSL4, 5‐LOX, and ALOX12. (2) NcRNAs in classical signaling pathways. NcRNAs regulate ferroptosis by targeting the p53/NRF2–SLC7A11–GPX4 axis. (3) NcRNAs in iron metabolism. NcRNAs regulate ferroptosis by targeting iron metabolism‐related molecules such as ferroportin, ferritin, TF, and TFR. 5‐LOX, 5‐lipoxygenase; ACSL4, acyl‐CoA synthetase long‐chain family member 4; ALOX12, arachidonate 12‐lipooxygenase; NcRNA, noncoding RNA; NRF2, nuclear factor erythroid 2‐related factor 2; TF, transferrin; TFR, transferrin receptor.

Notably, a miRNA does not act on a single gene. For example, miR‐7‐5p inhibits ferroptosis by upregulating the expression of ferritin and simultaneously downregulating the expression of ALOX12 and decreasing the level of LiperFluo.^139^ MiRNAs action is also coordinated with other epigenetic mechanisms to regulate ferroptosis. MiR‐34a‐5p downregulates the expression of SIRT1 to increase the sensitivity of cadmium‐induced ferroptosis,^140^ and miR‐522 protects against ferroptosis by downregulating ALOX15 expression facilitated by USP7.^141^ The latter process requires the assistance of heterogeneous nuclear ribonucleoprotein A1 (hnRNPA1) (Figure 5).

#### 3.3.2 Circular RNAs in ferroptosis

A circular RNA (circRNA) is a special ncRNA molecule that is back‐spliced from pre‐mRNA, and it is a recently growing research hotspot in the field of ncRNAs. Similar to those on miRNA, studies on the regulation of ferroptosis by circRNAs have been focused mainly on tumors, diabetes and ischemic diseases.^142^, ^143^, ^144^, ^145^, ^146^, ^147^, ^148^, ^149^, ^150^, ^151^, ^152^

CircRNAs, which belong to competing endogenous RNAs (ceRNAs), directly sponge miRNA via their miRNA‐binding sites, thereby eliminating the inhibitory effect of miRNAs on their target genes. Depending on the miRNA targeted by a circRNA, the regulatory effect on ferroptosis differs. Some tumor‐related genes have been confirmed to be directly or indirectly associated with ferroptosis via circRNA‐miRNA‐target molecule regulatory networks. CircRHOT1, circ‐0008035, circRNA1615, and circPSEN1 protect against ferroptosis via the miR‐106a‐5/STAT3, miR‐599/EIF4A1, miR152/LPR6, and miR‐200b‐3p/cofilin‐2 axes, respectively.^142^, ^143^, ^150^, ^152^ Additionally, in lipid metabolism, circPtpn14 positively regulates ferroptosis by targeting miR‐351‐5p, inhibiting the expression of 5‐LOX,^153^ while circDTL, circIL4R, circKIF4A, and cicr0000309 increase the expression of GPX4 by sponging miR‐1287‐5p, miR‐541‐3p, miR‐1231, and miR‐188‐3p respectively, inhibiting ferroptosis.^125^, ^144^, ^145^, ^151^ Notably, a single circRNA carries multiple miRNA‐binding sites. CircEPSTI1 inhibits ferroptosis by simultaneously sponging miR‐375, miR‐409‐3p, and miR‐515‐5p to upregulate SLC7A11 expression (Figure 5).^146^

In addition, circRNAs interact with RNA‐binding proteins to regulate gene expression. Hsa‐circ‐0008367 binds with ALKBH5, a negative regulator of autophagy, to promote ferroptosis.^147^ Notably, it has been proven that partial circRNAs show the potential to serve as a template for protein translation,^148^, ^149^, ^154^ and certain encoded proteins can active ferroptosis‐related signaling pathway^149^; however, whether circRNAs regulate ferroptosis through this mechanism remains to be confirmed.

#### 3.3.3 Long ncRNAs in ferroptosis

Long noncoding RNAs (lncRNAs) constitute a class of ncRNAs greater than 200 nucleotides in length. Many studies have shown that the abnormal expression of lncRNAs is closely related to the pathogenesis of diseases such as tumors, degenerative diseases and ischemic injury,^155^, ^156^, ^157^, ^158^ which have been proven to be associated with ferroptosis. On this basis, an increasing number of ferroptosis‐related lncRNAs have been discovered. Additionally, in different tumors such as lung adenocarcinoma (LUAD), breast cancer, colon cancer, and bladder cancer, ferroptosis‐related lncRNA signatures related to prognosis have been established via Cox regression analysis.^159^, ^160^, ^161^, ^162^

Mechanistically, lncRNAs mainly regulate ferroptosis through posttranscriptional processes. First, some lncRNAs regulate ferroptosis through a ceRNA network, which is similar to that of circRNA. LncRNA PVT1 actives *p53* gene expression through miR‐214 to induce ferroptosis.^158^ As a molecule downstream of p53, SLC7A11 is also regulated via the ceRNA network. The lncRNA OIP5‐AS1 and lncRNA SLC16A1‐AS1 separately upregulate SLC7A11 expression by targeting miR‐128‐3p and inhibit ferroptosis.^163^, ^164^ In addition to system Xc^−^, the ceRNA network targets another amino acid transporter. LncRNA ZFAS1 negatively regulates the expression of miR‐150‐5p, which targets SLC38A1, a regulator of glutamine absorption and metabolism, to induce ferroptosis.^165^ In addition, lncRNA participate in the regulation of the transsulfuration pathway, which is critical for the generation of cysteine. LncRNA LINC00336 interacts with ELAV‐like RNA‐binding protein 1 (ELAVL1) to inhibit miR6852, leading to cell resistance to ferroptosis by upregulating CBS expression.^166^ These abovementioned results suggested that lncRNAs are extensively involved in lipid metabolism in ferroptosis. Additionally, studies have identified lncRNAs as key mediators regulating iron metabolism during ferroptosis. LncRNA NEAT1 increases while lncRNA PR11‐89 decreases the cellular iron concentration to regulate ferroptosis, in which the former sponges miR‐9‐5p to upregulate the expression of TFR and GOT1 and the latter sponges miR‐129‐5p to upregulate the expression of PROM2.^167^, ^168^ Interestingly, a recent study found diametrically opposing regulation to that mediated by the ceRNA network, where lncRNAs stabilize the structure of miRNAs.^169^ This kind of regulation has also been found in relation to ferroptosis. For example, lncRNA MT1DP targets miR‐365a‐3p to downregulate the expression of NRF2, increasing cellular sensitivity to ferroptosis.^170^ It is necessary to find more lncRNAs similar to lncRNA MT1DP to complement the lncRNA‐miRNA regulatory network. Second, lncRNAs bind with mRNAs to regulate the translation process. LncRNA GABPB1‐AS1 directly inhibits the translation of *GABPB1* mRNA, which downregulates the expression of peroxiredoxin‐5 (PRDX5) to induce ferroptosis.^171^ Additionally, certain lncRNAs regulate mRNA translation via lncRNA‐protein complexes. The lncRNA 00925 binds with the pumilio RNA‐binding family member 2 (Pum2) protein, leading to the degradation of *Prdx6* mRNA,^172^ while the lncRNA ASMTL‐AS1 recruits U2AF2, which stabilizes the structure of *SAT1* mRNA to induce ferroptosis (Figure 5).^173^

In addition to the posttranscriptional regulation, lncRNAs directly or indirectly regulate the transcription of genes. LncRNA Meg3, located both in the nucleus and cytoplasm, directly binds to the *p53* gene and induces ferroptosis through the p53–GPX4 axis,^174^ while the cytosolic lncRNA P53RRA necessarily cooperates with ras GTPase‐activating protein‐binding protein 1 (GABP1) to active the *p53* gene.^175^ The regulation of genes by lncRNA LINC00618 also requires the participation of proteins. The lncRNA LINC00618 promotes ferroptosis in an apoptosis‐dependent manner and inhibits the expression of SLC7A11 by attenuating LSH, inducing the transcription of *SLC7A11* after recruitment to the *SLC7A11* promoter.^176^

Overall, ncRNAs can regulate ferroptosis in multiple ways, and the epigenetic mechanisms of ncRNA action are both interrelated and independent. Therefore, it is necessary to establish a complete lncRNA/circRNA–miRNA epigenetic regulatory network. In addition, many ncRNAs are located in knowledge blind spots, and how many of these ncRNAs are related to ferroptosis is unknown and deserves further exploration.

### 3.4 RNA modifications in ferroptosis

RNA methylation, which has become a hot topic in epigenetic research over the past decade, accounts for more than 60% of all RNA modifications, and among RNA methylation modifications, RNA m^6^A is the most prevalent posttranscription modification of mRNAs.^177^ The biological function of the RNA m^6^A modification depends on “writers,” “erasers,” and “readers.” The RNA m^6^A writers mainly include METTL3, METTL14, WTAP, and KIAA1429, which mediate the methylation of RNA. ALKBH5 and FTO are erasers, that are critical for the demethylation of RNA m^6^A. Readers, RNA m^6^A‐binding proteins, recognize mRNA with the m^6^A mark; the readers mainly include the YTH domain protein family and HNRNP family.^178^

Evidence suggests that the RNA m^6^A modification regulates ferroptosis. In non‐small cell lung carcinoma, FSP1, a glutathione‐independent ferroptosis inhibitor with an mRNA carrying five m^6^A sites, is upregulated by METTL3 sponging.^179^ This result has been confirmed by the latest research performed by our group. In the aortas of type A AD patients, the protein level of METTL3 was negatively correlated with the expression of FSP1. We also found that METTL3 inhibited the expression of SLC7A11 to promote ferroptosis in human aortic smooth muscle cells.^180^ However, in other diseases, such as hepatoblastoma and LUAD, METTL3 enhanced the stability of *SLC7A11* mRNA to suppress ferroptosis.^181^, ^182^ These diametrically opposed effects may depend on the differential expression of the reader. Generally, YTHDF1 can promote the translation of its target mRNA.^183^, ^184^, ^185^ For example, in hepatocellular carcinoma (HCC), YTHDF1 recognizes the m^6^A mark on *SLC7A11* mRNA to enhance the inhibition of ferroptosis,^182^ and in hepatic stellate cells, YTHDF1 also recognizes m^6^A mark on the *BECN1* mRNA, enhancing ferritinophagy to induce ferroptosis.^186^ However, another member of the YTH family, YTHDF2 exhibits biological effects opposite to YTHDF1 in that it causes mRNA's decay.^187^ A study coupled YTHDF2 and METTL14 and demonstrated that the degradation of *SLC7A11* mRNA by YTHDF2 requires the METTL14‐mediated RNA m^6^A modification in HCC.^188^ Additionally, in LUAD, YTHDC2 targets SLC3A2 and SLC7A11 in an RNA m^6^A‐dependent manner to act as an endogenous ferroptosis inducer (Figure 4B).^189^ Erasers are also involved in the regulation of ferroptosis. In hypopharyngeal squamous cell carcinoma, ALKBH5 targets RNA m^6^A residues in the 3′UTR of the *NRF2* transcript and mediates transcriptional repression to promote ferroptosis.^190^ In lung cells, black phosphorus quantum dots decreased the expression level of ALKBH5, which increased the global RNA m^6^A level, causing lipid peroxidation, iron overload and mitochondrial dysfunction.^187^ As another eraser, FTO downregulated the expression of SLC7A11 to induce ferroptosis in thyroid cancer.^191^ Moreover, the RNA m^6^A modification has also been linked to ncRNAs to regulate ferroptosis. In doxorubicin‐treated cardiomyocytes, METTL14 promoted the RNA m^6^A modification of the lncRNA KCNQ1OT1, which is a sponge of miR‐7‐5p. Deficiency in miR‐7‐5p induced ferroptosis by increasing the level of TFR.^192^

Although studies on the regulation of ferroptosis mediated by the RNA m^6^A modification have been carried out only in recent years, clearly, the recent research on RNA m^6^A modification is focused mostly on writers and readers, with few studies on erasers. In addition, RNA modification is not limited to the m^6^A mark, and other common RNA modifications such as 2′‐O‐methylation (Nm) and pseudouracil (ψ) may be directions of further research.

## 4 THE ROLE OF EPIGENETIC REGULATORS IN FERROPTOSIS‐RELATED BIOLOGICAL PROCESSES AND DISEASES

### 4.1 Ferroptosis‐related biological processes

The role of ferroptosis in biological process has always been discussed. Indeed, ferroptosis has been implicated to cancer since its discovery,^16^ and various tumor suppressors have been subsequently found to exhibit tumor inhibition effect through increasing cellular ferroptosis sensitivity. The role of tumor suppressor p53 in ferroptosis has been thoroughly investigated. Although there is ongoing debate regarding the precise role of p53 in regulating ferroptosis, it is widely believed that its primary role is to promote ferroptosis. As previously mentioned, p53 can suppress the occurrence of ferroptosis by regulating different targets including *SLC7A11*, *ALOX12*, *SAT1*, and *GLS2*.^10^, ^14^, ^58^, ^59^ Further research has indicated that p53‐mediated ferroptosis has been found to be sufficient for tumor suppression in animal model.^57^ Therefore, we can reasonably assume that ferroptosis plays a critical role in tumor suppression process. Additionally, immune function may be another potential biological effect of ferroptosis. Evidence shows that ferroptosis‐related molecules is regulated by various cytokines. Interferon‐γ inhibits the expression of system Xc^−^,^193^ while interleukins 4 and interleukins 13 upregulate the expression of ALOX15 with downregulating expression of GPX4.^194^ Moreover, it has been found that ferroptosis may participate in physiological process such as erythropoiesis and aging with the generation of an antibody (HNEJ‐1), which is responsible for the reorganization of 4‐hydroxy‐2‐nonenal (HNE).^195^

In conclusion, the tumor‐suppressing role of ferroptosis is well established, and the immunological functions of this process still require further confirmation. Furthermore, it remains to be determined whether ferroptosis is involved in other biological processes, and additional research is needed to fully address these questions.

### 4.2 Ferroptosis‐related diseases

Although the physiological function of ferroptosis has not been demonstrated clearly, numerous studies have shown the role of ferroptosis in a variety of diseases. With its physiological tumor suppression function, inhibition of ferroptosis can drive the development of cancer. A total analysis of pan‐cancer patients revealed higher expression level of GPX4 in tumor tissues than in normal tissues.^85^ It was also reported that selenoprotein is upregulated in tumor, which suggests the enhanced expression level of GPX4 in the development of tumor.^196^ These results demonstrated that inhibition of ferroptosis plays a critical role in tumorigenesis. Notably, the differential expression of GPX4 is not present in all types of tumors. In types including thymoma, bladder urothelial carcinoma, esophageal carcinoma, cervical/endocervical cancer, and skin cutaneous melanoma, GPX4 expression was not significantly higher than normal tissues.^85^ This result indicates that the presence of other differentially expressed ferroptosis‐related molecules, which are valuable to be explored as specific tumor biomarkers. Interestingly, it has been reported that ferroptosis may have a tumor induction effect. In pancreatic ductal adenocarcinoma, cancer cells undergoing ferroptosis released a type of damage‐associated molecular pattern named KRA^G12D^ to tumor microenvironment, which is subsequently internalized by macrophages via AGER pathway, inducing a shift in macrophages towards a pro‐tumor M2‐like phenotype and tumor growth.^197^ Therefore, it is essential to investigate the possible influence of the cellular microenvironment on the impact of ferroptosis in tumors.

In addition to cancer, ferroptosis is involved in non‐neoplastic diseases. Evidence has indicated that ferroptosis is a major contributor to the pathogenesis of ischemia/reperfusion injuries (IRI) in different organs including the kidney, heart, and liver. In *GPX4* knockdown mouse, induction of ferroptosis by depletion of GPX4 increased IRI‐mediated acute renal failure.^198^ Similarly, overexpression of the ATP‐releasing pathway protein pannexin 1 induced ferroptosis via the MAPK/ERK signaling pathway in mouse subjected to renal IRI.^199^ Conversely, inhibition of ferroptosis can alleviate organ injury caused by IRI. Ferroptosis inhibitors, such as iron chelators and glutaminolysis inhibitors, have been demonstrated to ameliorate IRI‐mediated cardiac injury in an ex vivo mouse heart model.^4^ This result was further confirmed by another in vivo study, which indicated that iron chelators and ferrostatin‐1 can reduce acute or chronic IRI‐mediated heart failure.^19^ Moreover, Liproxstatin‐1, a small molecule ferroptosis inhibitors, has been reported to be able to inhibit ferroptosis in IRI‐mediated hepatic damage in a preclinical model.^198^ Additionally, ferroptosis has been linked to a range of other diseases, such as liver and lung fibrosis, autoimmune diseases, and neurodegeneration.^26^, ^200^, ^201^, ^202^, ^203^ However, further research is necessary to confirm the causative role of ferroptosis in these conditions.

In general, the regulation of ferroptosis has the potential to provide substantial clinical advantages for relative biological disorders, and epigenetic modifications offer a promising approach in inducing or inhibiting ferroptosis.

### 4.3 The application of epigenetic regulators targeting ferroptosis in biological processes and diseases

Epigenetics play a role in biological processes related to ferroptosis, with tumor suppression being the main focus. One example of this involvement is the regulation of GPX4 gene expression. Specifically, increased levels of H3K4me3 and H3K27ac, as well as decreased levels of DNA methylation have been observed at the promoter region of *GPX4* gene in various tumors.^85^ This epigenetic regulation of *GPX4* gene suggested promising potential for the use of epigenetic regulators in the treatment of ferroptosis‐related diseases. Indeed, many epigenetic drugs or compounds have shown excellent effects in the therapy of ferroptosis‐related diseases (Table 1).

**TABLE 1:** Epigenetic drugs/compounds in ferroptosis‐related diseases therapy.

Drug/compounds	Target	Molecular mechanism	Effects on ferroptosis	Diseases	Stage of research	References
JQ1	BRD4 inhibitor	JQ1 induces the expression of SIRT1 and inhibits the expression of G9a to downregulate BRD4, decreasing the expression of GPX4 and SLC7A11	Promotion	Breast cancer; lung adenocarcinoma	In vivo	^69^
Ketamine	KAT5 inhibitor	Ketamine inhibits the expression of KAT5 to downregulate the expression of GPX4	Promotion	Breast cancer	In vitro	^72^
BEBT‐908	HDACs inhibitor	BEBT‐908 hyperacetylates p53 to upregulate its expression	Promotion	Colon adenocarcinoma	In vivo	^75^
Quisinostat	HDACs inhibitor	Unknown	Promotion	Tongue squamous cell carcinoma	In vivo	^76^
Vorinostat	HDACs inhibitor	Unknown	Promotion	Lung adenocarcinoma	In vitro	^77^
5‐aza‐CdR	DNMT1 inhibitor	5‐aza‐CdR decreases the methylation level of CDH1 to upregulate the expression of E‐cadherin	Inhibition	Head and neck cancer	In vitro	^65^
BRD4770	H3K9me 1/2/3 inhibitor	BRD4770 inhibits H3K9me1/2/3 abundance to upregulate the expression of FSP1, SLC7A11, GPX4, and GCH1	Inhibition	Aortic dissection	In vivo	^96^

Abbreviations: BRD4, bromodomain‐containing protein 4; CDH1, cadherin‐1; DNMT1, DNA methyltransferases 1; FSP1, ferroptosis suppressor protein 1; GCH1, GTP cyclohydrolase 1; GPX4, glutathione peroxidase 4; HDACs, histone deacetylases; KAT5, lysine acetyltransferase 5.

For different cancer, epigenetic regulators show potential of clinical application. In breast cancer, the BRD4 inhibitor JQ1 and the KAT5 inhibitor ketamine promote ferroptosis by epigenetic regulation of SIRT1/G9a and GPX4 respectively, and the former is also effective in LUAD.^69^, ^72^ Additionally, there are three HDAC inhibitors which show promise for treating cancer. In colon adenocarcinoma, BEBT‐908 promotes ferroptosis by hyperacetylating *p53*.^75^ Quisinostat and vorinostat inhibit tumor growth in tongue squamous cell carcinoma and lung adenocarcinoma, respectively, with unclear epigenetic regulation mechanism.^76^, ^77^ In addition to histone acetylation modification, DNA methylation is a potential target of epigenetic drugs for cancer treatment. In head and neck cancer, the DNMT1 inhibitor 5‐aza‐CdR inhibits ferroptosis by the epigenetic reprogramming of EMT,^65^ which provides a therapeutic approach for therapy‐resistant cancer. Moreover, epigenetic drugs have shown their clinical value not only in cancer, but also in AD. The antiferroptosis effects of BRD4770 by inhibiting H3K9me1/2/3 may reveal a potential therapeutic strategy for targeting VSMCs ferroptosis in AD.^96^

In conclusion, epigenetic drugs targeting ferroptosis provide more treatment options for related diseases. However, there are only a few epigenetic regulators that have clinical translational significance with diverse epigenetic modifications that efficiently regulate ferroptosis. Further research is needed to identify and develop more effective epigenetic drugs that can specifically target ferroptosis and improve treatment outcomes for related diseases.

## 5 CONCLUSIONS AND PERSPECTIVES

Since the concept of ferroptosis was first proposed, epigenetic regulation has become a hot topic in this field. Here, we summarize several types of epigenetic regulatory mechanisms in ferroptosis, such as histone modifications, DNA methylation, ncRNA, and RNA modifications. Undeniably, the epigenetic regulatory mechanisms in ferroptosis are complex and diverse, and targeting epigenetic marks is a promising strategy for the treatment of ferroptosis‐related diseases. However, before this possibility can be realized, many questions in epigenetic studies related to ferroptosis must be answered in the future.

First, recent research has been limited to a few key ferroptosis‐related molecules, such as GPX4 and SLC7A11. To determine whether epigenetic mechanisms regulate other FRGs, further exploration is needed. Organelle‐specific ferroptosis regulators are a valuable target. It has been found that ATF4 (a key molecular in the ER stress) and GLS2 (a key molecular in the mitochondria tricarboxylic acid cycle) are regulated by miRNA.^120^, ^126^ It is worth studying more interaction between epigenetics and organelle‐specific ferroptosis regulators. Additionally, the vast majority of ferroptosis‐related epigenetic mechanisms elucidated to date have been shown to regulate the expression of FRGs. However, the posttranslational modifications of these related genes including methylation, acetylation, and ubiquitination, which affect the activity, stability, and cellular localization of proteins, remain to be elucidated by more study. The dearth of investigation into these factors has limited our development of drugs targeting epigenetic modifications, which are important for interfering with ferroptosis and the treatment of ferroptosis‐related diseases.

Second, in ferroptosis, a single epigenetic regulation pathway is rarely active alone; instead, multiple pathways work together to regulate ferroptosis. On the one hand, multiple epigenetic modifications may regulate the same molecule. For example, high expression levels of GPX4 in tumor cells are simultaneously regulated by DNA methylation, histone methylation, and histone acetylation.^85^ On the other hand, a single epigenetic regulator such as BRD4770 and miR‐7‐5p can target multiple ferroptosis‐related molecules.^96^, ^139^ Thus, epigenetic regulatory pathways form a complex network to regulate ferroptosis. However, how different epigenetic modifications affect each other and the mechanisms that jointly participate in ferroptosis are largely unknown. In addition, a study has shown that histone acetylation mediated by PCAF is more likely to be involved in the regulation of transcription factors, serving as a fine‐tuning mechanism, not a switch.^204^ Whether this conclusion can be extended to other epigenetic regulatory mechanisms remains to be determined. Therefore, it is necessary to identify the major epigenetic regulatory mechanisms in ferroptosis, which may help us identify appropriate drugs with better targeting.

Finally, although some epigenetic regulators mentioned in this paper, such as, quisinostat, BRD4770, 5‐aza‐CdR, and miRNA nanomedicine, have shown excellent effects in the treatment of ferroptosis‐related diseases, each study has been limited to one disease model. Indeed, regulation of ferroptosis with the treatment of the same epigenetic modification can show opposite effects in different tissues or diseases.^78^, ^87^, ^88^, ^89^, ^96^, ^97^ The reasons for cell‐specific biological effects of epigenetic modifications are not clear, which may be influenced by multiple factors. In addition to the cell‐specific expression of ferroptosis‐related protein and cell microenvironment aforementioned, there is another factor to consider, which is the difference of the type and number of organelles in different cells, for example, mitochondria are most abundant in myocardial cells. The occurrence of ferroptosis depends on the integration of signals from different organelles including mitochondria, lysosomes, ER, and so on. Epigenetic modifications may lead to cell‐specific biological effects by regulating organelle‐specific ferroptosis‐related proteins. Overall, the cell‐specific biological effects of epigenetic modifications will be a valuable research direction. Furthermore, since the induction or inhibition of ferroptosis by epigenetic marks affects nontargeted cells, including normal cells, research on the cell‐specificity of ferroptosis mediated by epigenetic modulators may help prevent the side effects of drugs used in the clinic. Obviously, the exploration of these problems will be of great help for guiding drug use in different diseases.

## AUTHOR CONTRIBUTIONS

The contribution of the article confirmed as follows: M. Y. and H. L. wrote and edited the manuscript; X. Y. searched and collected literatures; X. W. and D. J. provided ideas and funding support. All authors approved the article for publication.

## CONFLICT OF INTEREST STATEMENT

The authors declare that they have no competing interests.

## ETHICS STATEMENT

Not applicable.

## FUNDING INFORMATION

This work was supported by grants from the National Natural Science Foundation of China (No. 82170502 and No. 82070488).

## Data Availability

Not applicable.
